# Chronic conditions, immunosuppression, and delayed and missed care during the COVID-19 pandemic (2020-2021)

**DOI:** 10.1093/geroni/igaf122.3334

**Published:** 2025-12-31

**Authors:** Natalie Turner, Melissa Martinson

**Affiliations:** University of Washington, Seattle, Washington, United States; University of Washington, Seattle, Washington, United States

## Abstract

The COVID-19 pandemic severely disrupted healthcare provision, leading many to delay or miss medical care. Within the first few months, one-third of adults age 65 and older reported delaying or missing care. Additionally, individuals with chronic conditions faced heightened barriers to accessing healthcare. While some research has examined age-related differences in healthcare access, less is known about how age interacts with specific chronic conditions regarding delayed or missed care. This study examined the role of age in delayed or missed medical care using the National Health Interview Survey 2020–2021 waves. Multivariate logistic regression models assessed the relationship between age, chronic illness, and healthcare disruptions, while exploring disparities by race, ethnicity, income, and insurance status. Adults age 65 and older were less likely to delay or miss care than those age 18–44 and 45-64. These findings remained consistent among individuals with hypertension, cardiovascular disease, and lung disease. However, among immunocompromised individuals and those with diabetes, age was not associated with delayed or missed care. Black and Hispanic individuals with diabetes, and American Indian and Alaska Native individuals with cardiovascular disease experienced greater healthcare disruptions. Immunocompromised individuals with the lowest incomes had increased likelihood of delaying or missing care. These results suggest that while older adults generally experienced fewer disruptions in healthcare, certain chronic conditions may pose unique challenges regardless of age. Findings also highlight how racial disparities in access to care vary across chronic conditions. Future research should examine how outreach efforts, including telehealth, may mitigate barriers to healthcare.

